# Can wood-decaying urban macrofungi be identified by using fuzzy interference system? An example in Central European *Ganoderma* species

**DOI:** 10.1038/s41598-021-92237-5

**Published:** 2021-06-24

**Authors:** Alžbeta Michalíková, Terézia Beck, Ján Gáper, Peter Pristaš, Svetlana Gáperová

**Affiliations:** 1 Department of Computer Sciences, Faculty of Natural Sciences, Matej Bel University, 97401 Banská Bystrica, Slovak Republic; 2 Department of Biology and Ecology, Faculty of Natural Sciences, Matej Bel University, 97401 Banská Bystrica, Slovak Republic; 3 Department of Biology and Ecology, Faculty of Ecology and Environmental Sciences, Technical University in Zvolen, 96001 Zvolen, Slovak Republic; 4 Department of Biology and Ecology, Faculty of Sciences, University of Ostrava, 70103 Ostrava, Czech Republic; 5 Institute of Biology and Ecology, Faculty of Sciences, Pavol Jozef Šafárik University in Košice, Šrobárova 2, 04001 Košice, Slovak Republic

**Keywords:** Computational biology and bioinformatics, Systems biology

## Abstract

*Ganoderma* is a cosmopolitan genus of wood-decaying basidiomycetous macrofungi that can rot the roots and/or lower trunk. Among the standing trees, their presence often indicates that a hazard assessment may be necessary. These bracket fungi are commonly known for the crust-like upper surfaces of their basidiocarps and formation of white rot. Six species occur in central European urban habitats. Several of them, such as *Ganoderma adspersum*, *G*. *applanatum*, *G*. *resinaceum* and *G*. *pfeifferi*, are most hazardous fungi causing extensive horizontal stem decay in urban trees. Therefore, their early identification is crucial for correct management of trees. In this paper, a fast technique is tested for the determination of phytopathologically important urban macrofungi using fuzzy interference system of Sugeno type based on 13 selected traits of 72 basidiocarps of six *Ganoderma* species and compared to the ITS sequence based determination. Basidiocarps features were processed for the following situations: At first, the FIS of Sugeno 2 type (without basidiospore sizes) was used and 57 *Ganoderma* basidiocarps (79.17%) were correctly determined. Determination success increased to 96.61% after selecting basidiocarps with critical values (15 basidiocarps). These undeterminable basidiocarps must be analyzed by molecular methods. In a case, that basidiospore sizes of some basidiocarps were known, a combination of Sugeno 1 (31 basidiocarps with known basidiospore size) and Sugeno 2 (41 basidiocarps with unknown basidiospore size) was used. 84.72% of *Ganoderma* basidiocarps were correctly identified. Determination success increased to 96.83% after selecting basidiocarps with critical values (11 basidiocarps).

## Introduction

*Ganoderma* P. Karst. (Ganodermataeae, Basidiomycota) is a cosmopolitan genus with the greatest diversity in the tropical regions^1^. These bracket fungi grow annually or can be perennial, sessile or stipitate, with dull or a laccate pileus surface and are commonly known by mycologists for the cream to dark purplish-brown-colored context, creamed-colored pore surface brownish after touching, ovoid, echinulate basidiospores with a truncate apex, two layered wall and interwall pillars between endosporium and exosporium^2–7^. Seven species occur in Central Europe^2,6–8^. *Ganoderma valesiacum* Boud. was omitted from the study because it is extremely rare. The species selected included *G*. *carnosum* Pat., *G*. *lucidum* s. str. (Curtis) P. Karst., *G*. *adspersum* (Schulzer) Donk, *G*. *applanatum* (Pers.) Pat., *G*. *pfeifferi* Bres. and *G*. *resinaceum* Boud. Especially the last four mentioned species cause white rot of living deciduous and coniferous trees of the wide range of species^9^. Plant pathologists and arborists know them collectively as a cause of decay in a very wide range of urban woody plant taxa^10,11^. Among standing trees, their presence is often taken to indicate that a hazard assessment may be necessary^11,12^. Therefore, their early identification is crucial for correct management of trees in urban landscapes^13–15^.

Several *Ganoderma* species are however difficult to distinguish based solely on morphological features and recently, molecular data have been used for *Ganoderma* spp. identification^16^. Molecular identification of fungal species relies mainly on DNA sequencing of ITS (internal transcribed spacer) region that is widely accepted as the “gold standard” by fungal taxonomists^17^. Schmidt et al.^18^ identified *G*. *applanatum* and *G*. *australe* at first from fruit-bodies grown on urban trees in Germany by ITS sequencing and subsequently also from the neighbouring rotten wood. Thus, an advantage of ITS sequencing is its ability to identify fungal species also if only mycelium or even only rotten wood are available. However, molecular methods tend to require expensive, stationary equipment that requires specialized skills to operate and fungi identification can be time-consuming^19^.

The aim of the present study is to develop a fast and reliable method for determining *Ganoderma* species based on selected qualitative and quantitative characters of the basidiocarps. Mathematical processing via fuzzy interference system of these characters can be done within 1 day.

From the mathematical point of view, the determination of some elements into some special classes is a problem of cluster analysis and classification. There are two main ways how to divide elements into clusters^20–23^. The first one is the use of hierarchical cluster analysis, which is based on statistical data processing. In the current big data era, where it is time-consuming or impossible to calculate all common character properties, the second one, non-hierarchical cluster analysis, is often used. Some of the non-hierarchical cluster analyses use soft computing tools as fuzzy sets or neural networks^24,25^. These systems and their combinations have been used to solve different biological problems^26–31^. The advantages of these methods can be viewed in the fast obtaining of the results. Moreover, they provide us with information about the existence of such elements which belong simultaneously to different clusters with certain degree (membership degree). This could help researchers to find the problematic/atypical items (usually when the membership degrees for more clusters are the same).

In this study, the non-statistical access by using fuzzy sets was chosen. Fuzzy sets are special mathematical structures that were developed for computing not just with the numbers but also with the words, which are used in everyday life. Since selected basidiocarp traits could be easily described using fuzzy sets, we focused on building a fuzzy inference system of the Sugeno type (Sugeno-type FIS) with constant output^32,33^, so that if specific trait values are defined as inputs, the species of *Ganoderma* will be generated as output by the Sugeno-type FIS.

The FIS of the Sugeno type with constant output consist of the rules which have the following form

$${R}_{i}: \mathrm{I}\mathrm{f} \left({X}_{1} \mathrm{i}\mathrm{s}\, {A}_{1i}\right)\,\,\mathrm{a}\mathrm{n}\mathrm{d} \left({X}_{2}\, \mathrm{i}\mathrm{s}\, {A}_{2i}\right)\,\,\mathrm{a}\mathrm{n}\mathrm{d} \dots \mathrm{a}\mathrm{n}\mathrm{d}\, \left({X}_{n}\, \mathrm{i}\mathrm{s}\, {A}_{ni}\right), \quad \mathrm{t}\mathrm{h}\mathrm{e}\mathrm{n}\, (Y\, \mathrm{i}\mathrm{s}\, {b}_{i}) .$$

In this form $R$ represent the rule, $i$ represent the rules order, ${X}_{1}, {X}_{2}, \ldots, {X}_{n}$ represent input linguistic variables, ${A}_{1i}, {A}_{2i}, \ldots, {A}_{ni}$ are the values of input linguistic variables defined by the fuzzy sets, $n$ represent the number of input linguistic variables, $Y$ represent an output linguistic variable and ${b}_{i}$ is the value of output linguistic variable defined by the constant. Let's have the particular numerical values of the characters, $\boldsymbol{x}\boldsymbol{ }= ({x}_{1}, {x}_{2},\ldots , {x}_{n})$ and let the system consist of $k$ rules. For each rule ${R}_{i}$ and each particular numerical values of the characters $\boldsymbol{x}$ the weight of the rule ${w}_{i}$ is computed by the formula

$${w}_{i}=\underset{j=1,\dots ,n}{\mathrm{min}}\left({\mu }_{{A}_{ij}}\left({x}_{j}\right)\right) .$$

Moreover, there is a possibility to decide for each rule the power of the rule for the produced system. Denote this power weight by $\stackrel{-}{{w}_{i}}$. Since each rule ${R}_{i}$ had assigned the constant output ${b}_{i}$, then the final output for the specific numerical values of the character $\boldsymbol{x}$ is computed by the formula^33^

$${y}_{{\mathbb{_X}}}=\frac{\sum _{i=1}^{k}{w}_{i}\stackrel{-}{{w}_{i}}{b}_{i}}{\sum _{i=1}^{k}{w}_{i}\stackrel{-}{{w}_{i}}} .$$

## Materials and methods

In total, 72 basidiocarps of six *Ganoderma* species collected in Central European region, identified by anatomo-morphological and molecular data^39^, were evaluated. Identified species include *G*. *adspersum* (19 basidiocarps), *G*. *applanatum* (27 basidiocarps), *G*. *carnosum* (9 basidiocarps), *G*. *lucidum* s. str. (4 basidiocarps), *G*. *pfeifferi* (7 basidiocarps) and *G*. *resinaceum* (6 basidiocarps). Twenty-one woody plant taxa, namely *Acer negundo* L., *Acer platanoides* L., *Acer pseudoplatanus* L., *Acer* sp., *Aesculus hippocastanum* L., *Alnus* sp., *Fagus sylvatica* L., *Fraxinus excelsior* L., *Gleditsia triacanthos* L., *Larix decidua* Mill.*, Picea* sp.*, Pinus nigra* J. F. Arnold*, Populus tremula* L., *Populus* sp., *Quercus petraea* (Matt.) Liebl., *Quercus robur* L., *Quercus* sp., *Salix alba* L., *Tilia cordata* Mill., *Tilia platyphyllos* Scop. and *Tilia* sp., were identified as hosts for the *Ganoderma* species. The fungal specimens are deposited in the Herbarium of the Department of Biology and Ecology, Faculty of Natural Sciences, Matej Bel University in Banská Bystrica, Slovakia. Nomenclature and authorities are from Index Fungorum (Cooper and Kirk 40) for fungi and The Plant List^35^ for woody plants.

The aim of this study was to create the FIS of the Sugeno type with constant output in such a way that the given values of the characters are defined as inputs, the particular *Ganoderma* species will be generated as output by the FIS. In the first step the input and output variables with their parameters need to be defined. The selected traits of the basidiocarps were described by *13* characters (*n* = *13*) designated as *X*_*1*_ to *X*_*13*_ Table 1. Each of these characters was designated as one linguistic variable input and the values of linguistic variables were assigned according to the real situation. The values of input linguistic variables were defined by the fuzzy sets. Each fuzzy set was exactly defined by its membership function. The data were processed in the program MATLAB^34^. For easily processing of the characters, the software application was developed in the program MATLAB Runtime Library^36^. In this program, trapezoidal functions are given by the four [*a, b, c, d*] where *a, b, c, d* are real numbers. The list of the used parameters of input linguistic variables is shown in Table 2.

**Table 1:** Summary table of characters and characteristics of the studied *Ganoderma* basidiocarps.

Character	Characteristic of character studied
*X*_*1*_/basidiospore length without exosporium (mean, µm)	The individual value indicates the arithmetic mean of 30 replications
*X*_*2*_/basidiospore width without exosporium (mean, µm)	the individual value indicates the arithmetic mean of 30 replications
*X*_*3*_/stem presence	0 no/1 yes
*X*_*4*_/pileus surface	0 crusty/ 1 shiny
*X*_*5*_/resinous crust presence	0 no/ 1 yes
*X*_*6*_/pileus shape	0 ungulate 0.5 no well-defined or deformed 1 flat
*X*_*7*_/pileus surface colour	0 brown 1 dark brown
*X*_*8*_/pileus surface colour–surface pattern	1 beige–grey–brown beige–grey beige–grey brown 2 fawn brown–buff–clay brown–green brown–olive brown 3 ochre yellow, orange brown–pearl orange–ochre brown–red orange 4 chocolate brown–nut brown–chestnut brown 5 red brown–mahogany brown–purple violet 6 black red–jet black
*X*_*9*_/stem colour, if present	0 pure red–red brown–mahogany brown 0.5 stem absent 1 chocolate brown–red black
*X*_*10*_/tube layer colour	1 beige–sand yellow 2 brown beige–ivory 3 ochre brown 4 signal brown–fawn brown 5 nut brown–chestnut brown–clay brown
*X*_*11*_/pileus margin	0 round 0.5 not well-defined 1 sharp
*X*_*12*_/basidiocarp weight	0 not very light in weight 1 very light in weight
*X*_*13*_/context colour	1 from beige to almost sand yellow 2 sand yellow–fawn brown 3 beige brown–brown beige, orange brown 4 orange brown–ochre brown 5 nut brown–clay brown 6 nut brown–chestnut brown–mahogany brown
*Y*/species	identified by anatomo-morphological and molecular data

**Table 2:** List of the used parameters of input linguistic variables.

Studied parameters (= input linguistic variables)—used definition scope	Used values of input variables (= the names of the fuzzy sets)	Parameters of the used fuzzy sets
*X*_*1*_/basidiospore length without exosporium [5.0 9.5]	Low length of basidiospore without exosporium Middle length of basidiospore without exosporium High length of basidiospore without exosporium	[6.0 7.0 8.8 9.3] [8.8 9.3 10.4 13.0] [10.0 11.5 13.0 14.0]
*X*_*2*_/basidiospore width without exosporium—[7.0 13.0]	Low length of basidiospore without exosporium Middle length of basidiospore without exosporium High length of basidiospore without exosporium	[4:0 5.0 6.0 6.2] [6.0 6.2 7.4 8.5] [7.3 7.9 9.5 10.0]
*X*_*3*_/stem presence—[0.0 1.0]	Presence of stem Absence of stem	[− 0.2 − 0.1 0.1 0.9] [0.1 0.9 1.1 1.2]
*X*_*4*_/pileus surface—[0.0 1.0]	Crusty pileus surface Shiny pileus surface	[− 0.2 − 0.1 0.1 0.9] [0.1 0.9 1.1 1.2]
*X*_*5*_/resinous crust presence—[0.0 1.0]	Presence of resinous crust Absence of resinous crust	[− 0.2 − 0.1 0.1 0.9] [0.1 0.9 1.1 1.2]
*X*_*6*_/pileus shape—[0.0 1.0]	Ungulate pileus shape Flat pileus shape	[− 0.2 − 0.1 0.1 0.9] [0.1 0.9 1.1 1.2]
*X*_*7*_/pileus surface colour—[0.0 1.0]	Brown pileus surface colour Dark brown pileus surface colour	[− 0.2 − 0.1 0.1 0.9] [0.1 0.9 1.1 1.2]
*X*_*8*_/pileus surface colour—surface pattern—[0.0 7.0]	Beige–grey–brown beige–grey beige–grey brown surface pattern Fawn brown–buff–clay brown–green brown–olive brown surface pattern Ochre yellow, orange brown–pearl orange–ochre brown–red orange surface pattern Chocolate brown–nut brown–chestnut brown surface pattern Red brown–mahogany brown–purple violet surface pattern Black red–jet black surface pattern	[0.0 1.0 1.0 2.0] [1.0 2.0 2.0 3.0] [2.0 3.0 3.0 4.0] [3.0 4.0 4.0 5.0] [4.0 5.0 5.0 6.0] [5.0 6.0 6.0 7.0]
*X*_*9*_/stem colour, if present—[0.0 1.0]	Pure red–red brown–mahogany brown stem colour Chocolate brown–red black stem colour	[− 0.2 − 0.1 0.1 0.9] [0.1 0.9 1.1 1.2]
*X*_*10*_/tube layer colour—[0.0 6.0]	Beige–sand yellow tube layer colour Brown beige–ivory tube layer colour Ochre brown tube layer colour Signal brown–fawn brown tube layer colour Nut brown–chestnut brown–clay brown tube layer colour	[0.0 1.0 1.0 2.0] [1.0 2.0 2.0 3.0] [2.0 3.0 3.0 4.0] [3.0 4.0 4.0 5.0] [4.0 5.0 5.0 6.0]
*X*_*11*_/pileus margin—[0.0 1.0]	Round pileus margin Sharp pileus margin	[− 0.2 − 0.1 0.1 0.9] [0.1 0.9 1.1 1.2]
*X*_*12*_/basidiocarp weight—[0.0 1.0]	Not very light basidiocarp in weight Very light basidiocarp in weight	[− 0.2 − 0.1 0.1 0.9] [0.1 0.9 1.1 1.2]
*X*_*13*_/context colour—[0.0 7.0]	From beige to almost sand yellow context colour Sand yellow–fawn brown context colour Beige brown–brown beige, orange brown context colour Orange brown–ochre brown context colour Nut brown–clay brown context colour Nut brown–chestnut brown–mahogany brown context colour	[0.0 1.0 1.0 2.0] [1.0 2.0 2.0 3.0] [2.0 3.0 3.0 4.0] [3.0 4.0 4.0 5.0] [4.0 5.0 5.0 6.0] [5.0 6.0 6.0 7.0]

*X*_*1*_*, X*_*2*_ characters: light microscopic analysis of basidiospores was performed on fertile basidiocarps. The basidiospores were mounted in 5% KOH with cotton blue. Their size, without exosporium and without expanded vesicular apex, was measured with maximum magnification (with immerse objective 100×) of a MOTIC light microscope (MOTIC Company, Germany). The width and length of 30 basidiospores from each fertile basidiocarp were measured. The individual value indicates the arithmetic mean of 30 replications.

*X*_*3*_*, …, X*_*8*_ morphological characters of basidiocarps were described according to standard anatomo-morphological data^2,3,6,7,37^ and our characters of 72 basidiocarps.

*X*_*9*_*, …, X*_*13*_ colour characters of basidiocarps were measured using the List of RAL colours (RAL 840-HR)^38^.

*Y*: All *Ganoderma* basidiocarps were identified by the above-mentioned standard anatomo-morphological and molecular data. The total genomic DNA was isolated from basidiocarps, their ITS regions were amplified by PCR and sequenced in both directions at SEQme s.r.o. (Dobříš, Czech Republic). Obtained sequences have been deposited in GenBank database^39^.

As the output variables, one of the six *Ganoderma* species was expected. Since FIS of the Sugeno type with constant output allows to define each output variable as a constant, this type of FIS was used. The constants need to be defined on some interval and they have to be defined as the numbers. In this study, the universe *U* = [0, 1] was chosen. It is well known that several *Ganoderma* species have very similar characters, therefore the labelling of the species as it is mentioned in Table 3 was suggested.

**Table 3:** The list of the used values of output linguistic variables.

Ganoderma species	Assigned value of output
*G*. *adspersum*	1.0
*G*. *applanatum*	0.8
*G*. *carnosum*	0.6
*G*. *lucidum* s. str	0.4
*G*. *pfeifferi*	0.2
*G*. *resinaceum*	0.0

Since the parameters of input and output linguistic variables were designed, the fuzzy rules could be created. In the first step, the combinations of characters which are typical for each species need to be extracted. These combinations were extracted from both characters as they were defined in Table 1^2,3,6^. Since they are typical for each *Ganoderma* species, these types of rules were assigned the specific power weight which equals 1 in proposed FIS. In Example 1, there is indicated one of the used rules in such a form as it was defined in program MATLAB.

### *Example 1*

Example of the one of the used rules.

If (Basidiospore length without exosporium is Middle) and (Basidiospore width without exosporium is Middle) and (Basidiocarp without stem) and (Pileus surface is crusty) and (Pileus surface without resinous crust) and (Pileus shape is ungulate) and (Pileus surface colour/surface pattern is fawn brown/buff/clay brown/green brown/olive brown) and (Stem colour none) and (Tube layer colour is nut brown/chestnut brown/clay brown) and (Context colour is orange brown/ochre brown) and (Basidiocarp weight is not very light in weight) then (Species is *Ganoderma adspersum*) (power weight 1).

In our collection of 72 basidiocarps, there were visible also combinations of characters, mostly the shade of colours, in another form as it was mentioned in literature. We have also used the rules with these characters. Since these combinations of characters were not mentioned in the literature, these rules got power weight less than 1 (usually 0.75, 0.5, 0.25). The value of power weight depended on the number of variables in which the characters did not match the literature.

All the obtained rules were processed by program MATLAB in the Fuzzy Logic Designer Toolbox. Finally, FIS of the Sugeno type with constant output was created. It consisted of 243 fuzzy rules, which described the whole system and it was named as *Sugeno1.*

If someone wants to use a developed system, he/she needs to assign each of 13 characters to the particular values following the values mentioned in Table 1 (see Example 2).

### *Example 2*

One item from collection and its input values.

Table 4 represents the input values of one item from the collection. Therefore, for the vector of input values it hold

**Table 4:** Parameters of one item from the collection.

Studied parameters	Item parameters	Values of input value
Collection code	MS137	–-
*X*_*1*_/basidiospore length without exosporium (mean, µm)	10.9	*x*_*1*_ = 10.9
*X*_*2*_/basidiospore width without exosporium (mean, µm)	7.8	*x*_*2*_ = 7.8
*X*_*3*_/stem presence	No	*x*_*3*_ = 0.0
*X*_*4*_/pileus surface	Crusty	*x*_*4*_ = 0.0
*X*_*5*_/resinous crust presence	No	*x*_*5*_ = 0.0
*X*_*6*_/pileus shape	Ungulate	*x*_*6*_ = 0.0
*X*_*7*_/pileus surface colour	Brown	*x*_*7*_ = 0.0
*X*_*8*_/pileus surface colour–surface pattern	Fawn brown	*x*_*8*_ = 2.0
*X*_*9*_/stem colour, if present	Stem absent	*x*_*9*_ = 0.5
*X*_*10*_/tube layer colour	Nut brown	*x*_*10*_ = 5.0
*X*_*11*_/pileus margin	Round	*x*_*11*_ = 0.0
*X*_*12*_/basidiocarp weight	No very light in weight	*x*_*12*_ = 0.0
*X*_*13*_/context colour	Orange brown	*x*_*13*_ = 4.0
*Y*/species	*adspersum*	–

$$\boldsymbol{x} = ({x}_{1}, {x}_{2},\dots , {x}_{13}) = (10.9, 7.8, 0.0, 0.0, 0.0, 0.0, 0.0, 2.0, 0.5, 5.0, 0.0, 0.0, 4.0).$$

After the completing of particular numerical values of the characters *x*_*1*_*, x*_*2*_*, …, x*_*13*_ into the FIS, the system computes the output by the process, which was mentioned above. The output of the FIS was particular value from interval $[0, 1]$*.* There exist more approaches how to assign specific *Ganoderma* species considering the calculated value. The greatest problem is, when the output value is one of the values $0.9, 0.7, 0.5, 0.3$ and $0.1$ (critical values). For these output values, the system is not able to decide between two closely related *Ganoderma* species. We have decided to group them into a specific category: unrecognised. It could help us to find the critical individuals. According to the output values (except for critical values), we are able to decide upon a specific *Ganoderma* species. The insertion of *Ganoderma* species to particular output value is listed in Table 5.

**Table 5:** Insertion of *Ganoderma* species to particular output value.

*Ganoderma* species	Intervals without critical values
*G. adspersum*	(0.9, 1.0]
*G. applanatum*	(0.7, 0.9)
*G. carnosum*	(0.5, 0.7)
*G. lucidum* s. str	(0.3, 0.5)
*G. pfeifferi*	(0.1, 0.3)
*G. resinaceum*	[0.0, 0.1)

### *Example 3*

In the Example 2 input values of basidiocarp with collection code MS137 were mentioned. After insertion of these values into FIS, the system gave the result 1.0. This value belongs to the interval mentioned in the first row of the Table 5 therefore this basidiocarp is recognized as *G. adspersum*.

### *Example 4*

If the FIS output value of some basidiocarp reach, for example the value 0.9, then the basidiocarp will not be assigned to any *Ganoderma* species.

The insertion of each of 72 basidiocarps of this study is presented in the section “Results and discussion”.

However, only 31 fertile basidiocarps have been observed at the time of our collection, while the rest of the basidiocarps were sterile. Therefore, there was not possibility to determine the first two characters “Basidiospore length” and “Basidiospore width” in proposed FIS *Sugeno1* for all basidiocarps. For this reason, another FIS of the Sugeno type with constant output (named *Sugeno2*) was created. System *Sugeno2* consists of 11 input linguistic variables, except “Basidiospore length” and “Basidiospore width”. All other characters from FIS *Sugeno1* were used. The 41 sterile basidiocarps were processed by this system. The second system had also 243 rules. The process of calculation of the output values was the same as in the FIS *Sugeno1,* the only one difference was, that in the FIS *Sugeno2,* the user need to enter just 11 numerical values of the characters ${x}_{3}, {x}_{4}, \dots , {x}_{13}$ into the FIS. Subsequently, system calculates one specific value from interval $[0, 1].$ To assign the particular *Ganoderma* species considering to calculated output value, the values from Table 5 were used.

Let’s look at the created systems from the informatics point of view. FIS *Sugeno1* and FIS *Sugeno2*, which were created in program MATLAB, allows user to process characters of more basidiocarps at the same time. The user needs to prepare data as a matrix of characters in the right order. Each character need to be evaluated by a number, as it was mentioned in Table 1 and showed in Example 2.

The proposed system was developed on the basis of requirements of biological sciences. Since the filling of the character values as they were mentioned in Example 2 into the program MATLAB was not very meaningful from the biological point of view, also the software application was created. Proposed application consists of buttons, popup-menus and strings. They consist the characters in the natural form (as they were mentioned in Table 1, what means that user choose from the items expressed by the words (for example for “pileus surface colour–surface pattern” one of the possibilities is “beige–grey–brown beige–grey beige–grey brown”).

The characters, for which it is enough to decide between few “short” possibilities (yes/no, crusty/shiny) were defined by buttons. The characters, for which it is need to choose one “long” possibility (for “pileus surface colour–surface pattern” one of the possibilities “beige–grey–brown beige–grey beige–grey brown”) the popup-menus were used. The first button was designed as special button. It consists of the question if the scientist knows the measurements of the basidiospores without exosporium. If the answer is yes, then two strings are showed to user, to add the values of basidiospore length and width without exosporium, and the numeric values of them could be filled. If the answer is no, then the application consists just of the buttons and popup-menus. After the filling of all characters, the characters are transferred by the application to the numerical values mentioned in the Table 1 and processed by the designed FIS. Of course, the processing of the characters is also divided considering to known/unknown basidiospore measurements. If the scientist known these measurements, then the system uses FIS *Sugeno1*, if scientist didn´t know them, then the FIS *Sugeno2* is used.

After the filling of all characters, user just need to push the confirmation button and got as the result the classified *Ganoderma* species (for example “*Ganoderma adspersum*”)*.* When the value of result is not assigned to one specific *Ganoderma* species (it reach critical value), then the information “It is not possible to be exactly classified this basidiocarp” is displayed.

The application was developed such way that user did not need to have program MATLAB in the computer. It is enough to install MATLAB Runtime Library, which is free of charge. The application is suitable to classify the basidiocarps just one by one.

## Results and discussion

All 72 basidiocarps were identified using ITS sequence analysis with high similarity values. In general, 97% similarity cut-off (e.g.^41^) is applied for species delimitation in ITS analysis. At this level, clear separation of all but *Ganoderma carnosum/lucidum* species pair was observed. Several recent taxonomic revisions (e.g.^42^) that this cut-off score is too low for most fungi, and for some of tested *Ganoderma* species similarity values as high as 98.9% were observed.

Molecular based identification of *Ganoderma* specimens was then compared to the morphology-based identification using developed the FIS of type Sugeno with constant output. Similar to the molecular data the *Ganoderma* basidiocarps could be classified into the six classes (species). There are two possibilities how to use this FIS. The first one, directly in the program MATLAB, allows user to process characters of more basidiocarps together. The user needs to prepare data as a matrix of characters in the right order. Each character needs to be evaluated by a number, as it was mentioned in the Table 1. The second one, software application, allows user to process characters of basidiocarps one by one, but in this application the user define the characters of the basidiocarps by words, as they are really biologically specified. Since we had the collection of 72 basidiocarps, we used the application that was developed in program MATLAB.

Since the information of the basidiospore size for some basidiocarps was known and for some of them not, there were used two approaches. One without using of the information of basidiospore size for whole collection of basidiocarps. Second, where the information about basidiospore size was used for those basidiocarps from collection, for which it was known.

### Results obtained without using of the information of basidiospore size

In this part the basidiocarps features were used for species identification based just on morphological data. Each of 72 basidiocarps was defined by 11 characters and they were processed by using FIS *Sugeno2*. As an output system could give any value form the interval [0, 1]. For some basidiocarp the output value reach critical values (values 0.9, 0.7, 0.5, 0.3 and 0.1). The basidiocarps that did not reach critical values were classified into the classes due to intervals mentioned in Table 5. The basidiocarps that reached critical values were not classified into any class. By using this approach 57 basidiocarps were classified correctly Table 6. From 15 incorrectly identified basidiocarps the critical value was assigned to 13 basidiocarps Table 6—bold font).

**Table 6:** *Ganoderma* basidiocarps processed by FIS *Sugeno2.*

Species with their assigned value of output	Number of studied basidiocarps	Number of no classified = incorrectly identified basidiocarps	Code of incorrectly identified basidiocarps with their assigned value of output
*G. adspersum* 1.0	19	8	G2 0.8857 **MS137 0.9** **K29 0.9** G2ZH 0.8 **LV2 0.9** **GDS 0.9** **M124 0.5** **G13 0.5**
*G. applanatum* 0.8	27	3	**GVF 0.9** **G171 0.9** **K60 0.9**
*G. carnosum* 0.6	9	2	**SG4 0.5** **GCAND 0.5**
*G. lucidum* s. str. 0.4	4	1	**GLP 0.5**
*G. pfeifferi* 0.2	7	1	**M2 0.5**
*G. resinaceum* 0.0	6	0	–-
∑	72	15	

From used collection there were 15 incorrectly identified basidiocarps. The most of them were species of *G. adspersum* (8 basidiocarps) and *G. applanatum* (3 basidiocarps). From these 11 items for 7 items system gave as a result number 0.9, where 4 items with this value were *G*. *adspersum* and 3 items were *G*. *applanatum*. As it was mentioned before, the output values for these species were assigned as follows: *G. applanatum* interval (0.7, 0.9)*, G. adspersum* interval (0.9, 1.0]*.* This means, that system determined the incorrect classified items between these two species. However, in the field and herbaria, *G*. *applanatum* basidiocarps not attacked by larvae of *Agathomyia wankowiczii* (Diptera) or without both basidiospores and tube layers separated by thin layers of the context are externally almost indistinguishable from those of *G*. *adspersum*^39,43^. In addition, it should be noted that *G*. *applanatum* basidiocarps attacked by larvae occur irregularly as well as the first year of growth a perennial *Ganoderma* basidiocarps will have only one tube layer without thin layer of the context. Therefore, these characters could not be included in Table 1. In these cases, molecular studies based on the analysis of the DNA are needed to confirm the species determination and ITS sequence analysis clearly separated these two species with similarity level 90.52 only, significantly lower than required for species discrimination.

From the remaining 3 incorrectly identified items of *G*. *adspersum*, 1 item got as a result value 0.8857 which determined it again into the species *G*. *applanatum* and 2 of them reached the value 0.5 which determine these items somewhere between *G*. *carnosum* and *G*. *lucidum*.

Another 3 incorrectly identified items were species of *G*. *carnosum* (2 basidiocarps) and *G*. *lucidum* s. str. (1 basidiocarps). The situation with the results was similar as before. For all 3 items system gave as an output value number 0.5. The following output values were assigned to mentioned species: *G. lucidum* s. str. interval (0.3, 0.5)*, G. carnosum* interval (0.5, 0.7). This again means that system determined the items between these two species. Morphologically, mature *G*. *carnosum* basidiocarp has dark brown to black upper surface, usually grows on *Abies*, while *G*. *lucidum* s. str. has orange red to bay upper surface, usually grows on hardwoods^6^. However, in the field and herbaria, immature *G*. *carnosum* basidiocarps are superficially almost indistinguishable from those of *G*. *lucidum* s. str. Also, phylogenetically, sequences data from nuclear internal transcribed spacer regions (ITS) and the translation elongation factor 1-α gene (tef1-α) confirmed that *G*. *lucidum* groups together with *G*. *carnosum*^39,44^. Similarly, in our experiments similarity value 98.9% was observed between *G. carnosum* and *G. lucidum*, making this pair of species the most difficult for correct identification.

From seven basidiocarps of *Ganoderma pfeifferi*, 6 items got as an output value equal to 0.2 which mean that they were determined correctly and just one output value reached the value 0.5 and was determined incorrectly. No accurate items identification could be caused by untypical flat pileus shape (in this study), or by untypical dull basidiocarps of the otherwise laccate species *G*. *pfeifferi* in other studies^43^. The collection contains also 6 basidiocarps of *Ganoderma resinaceum*. All these items were determined by developed system correctly.

By using this method there were correctly classified 57 basidiocarps from all 72 basidiocarps. It could be calculated that the percentage of accurate classified basidiocarps reach the value 79.17%. It could be seen that this method assigned the critical value to 13 basidiocarps Table 6—bold font). System determines those basidiocarps, which need special attention from researcher. It means that researcher needs to process these items separately by using additional methods. If we omitted these items from the final table, then we could see that system makes mistakes only for 2 items from 59 and the percentage of accurate classified basidiocarps reach the value 96.61%.

### Results obtained with using of the information of basidiospore size

Among all basidiocarps tested, for 31 items, the information about basidiospore size was known. Therefore, we could use basidiocarps features for species identification based on morphological and morphometric data. In this step 31 items were processed by FIS *Sugeno1* and the rest 41 items we processed by FIS *Sugeno2.* The basidiocarps that did not reach critical values were classified into the classes due to intervals mentioned in Table 5. The basidiocarps which reached the critical value were labelled as incorrectly identified items. By using this approach 61 basidiocarps were classified correctly Table 7. From 11 incorrectly identified basidiocarps the critical value was assigned to 9 basidiocarps Table 7—bold font).

**Table 7:** No classified *Ganoderma* basidiocarps processed by FIS *Sugeno1* and *Sugeno2* among all basidiocarps tested.

Species with their assigned value of output	Number of fertile basidiocarps	Codes of fertile basidiocarps no classified by *Sugeno1* with their assigned value of output	Number of sterile basidio-carps	Codes of sterile basidiocarps no classified by *Sugeno2* with their assigned value of output	Number of all no classified basidiocarps
*G. adspersum* 1.0	9	**G13 0.5**	10	G2 0.8857 **K29 0.9** G2ZH 0.8 **MŠ124 0.5**	5
*G. applanatum* 0.8	8	–	19	**G171 0.9** **K60 0.9**	2
*G. carnosum* 0.6	4	**GCAND 0.5**	5	**SG4 0.5**	2
*G. lucidum* s. str. 0.4	4	**GLP 0.5**	0	–	1
*G. pfeifferi* 0.2	2	–	5	**M2 0.5**	1
*G. resinaceum* 0.0	4	–	2	–	0
∑	31		41		11

After the processing of basidiocarps by using the second access (combination of FIS *Sugeno1* and *Sugeno2*) we could conclude following results:

In the “Results obtained without using of the information of basidiospore size” from mentioned 11 incorrectly identified items of *G . adspersum* (8 basidiocarps) and *G . applanatum* (3 basidiocarps) there were 5 of them with the known information about the basidiospore size. The 4 from these 5 items were classified by second approach correctly. By this result we again confirmed the known fact 44 (and others) that the basidiospore length and width are important characters for determination of *G . adspersum* and *G . applanatum* . Nevertheless, that the information about the basidiospore size was known for 2 from 3 incorrectly classified items of *G . carnosum* (2) and *G . lucidum* s. str. (1), the situation did not improve after using combination of FIS Sugeno1 and Sugeno2. Morphologically, *G . carnosum* has ellipsoid basidiospores (10–13 × 7–8.5 μm), while *G . lucidum* s. str. has smaller ellipsoid (7–11 × 6–8 μm) basidiospores^6^. Other achieved results correspond well with the previous analysis of “Results obtained without using of the information of basidiospore size”. After this process the percentage increased on the value 84.72% Table 7 .

This method assigned the critical value to 9 basidiocarps. These basidiocarps need to be processed by additional method. If we omitted these 9 items from the final table, then we could see that system makes mistakes only for 2 items from 63 and the percentage of right classified basidiocarps reach the value 96.83%.

## Conclusions

The aim of this study was to develop a fast and reliable method for determination of European *Ganoderma* species based on selected qualitative and quantitative characters. 72 *Ganoderma* basidiocarps belonging to 6 species were determined using FIS of type Sugeno. At first, the FIS of type *Sugeno2* (without basidiospore sizes) was used to identify *Ganoderma* species and each tested basidiocarp was defined by 11 characters. 57 *Ganoderma* basidiocarps (79.17%) were correctly determined. Determination success increased to 96.61% after selecting basidiocarps with critical values (15 basidiocarps). These undeterminable basidiocarps must be analyzed by molecular methods.

In a case, that basidiospore sizes of some basidiocarps were known, a combination of Sugeno 1 (31 basidiocarps with known basidiospore size) and *Sugeno2* (41 basidiocarps with unknown basidiospore size) was used. 84.72% of *Ganoderma* basidiocarps were correctly identified. Determination success increased to 96.83% after selecting basidiocarps with critical values (11 basidiocarps). The basidiospore size data slightly increased the determination success of *Ganoderma adspersum*/*applanatum* basidiocarps by combining FIS of type *Sugeno1* and *Sugeno2*.

To our knowledge, this is the first application of fuzzy interference system of type Sugeno (Sugeno-type FIS) on the identification of macrofungi. This study confirmed the capabilities of Sugeno-type FIS as an effective practical technique for the determination of phytopathologically important urban macrofungi. At the basis of their features we classify collection of *Ganoderma* spp. specimens into the 6 classes in accordance with morphological and molecular species identification.

## Data Availability

The fungal specimens are deposited in the Herbarium of the Department of Biology and Ecology, Faculty of Natural Sciences, Matej Bel University in Banská Bystrica, Slovakia. Nomenclature and authorities are from Index Fungorum (Cooper and Kirk, 40) for fungi and The Plant List^35^ for woody plants. Molecular identification of fungal species relies mainly on DNA sequencing of ITS (internal transcribed spacer) region that is widely accepted as the “gold standard” by fungal taxonomists^17^.

## Code availability

Software application in MATLAB with functions was designed by authors.
